# Advanced Abdominal Pregnancy (AAP) after 20 Weeks of Gestation in Japan: A Retrospective Review

**DOI:** 10.1155/2021/6624404

**Published:** 2021-07-26

**Authors:** Tatsuji Hoshino, Tatsuo Mori, Yu Fujii, Shinya Yoshioka

**Affiliations:** ^1^Department of Obstetrics and Gynecology, Meiwa General Hospital, Nishinomiya, Japan; ^2^Department of Obstetrics and Gynecology, Kobe City Medical Center General Hospital, Kobe, Japan

## Abstract

**Background:**

An advanced abdominal pregnancy (AAP) rarely continues to a live birth, but sometimes, a live birth may occur. In developed countries, women with AAP who have not been diagnosed preoperatively are expected to be diagnosed quickly, and the pregnant woman and the fetus will be saved. After careful examination of the past cases, we sought to derive what is the best diagnosis and treatment choice in the current medical environment.

**Materials and Methods:**

We retrospectively studied AAP cases in Japan. We examined diagnosis of AAP before fetal delivery and placental treatment at the time of delivery. AAP was well documented in 10 cases. We contacted the AAP authors, who reported 10 AAP cases in Japan, directly to confirm any unclear points.

**Results:**

Two cases were diagnosed with AAP before laparotomy, one was diagnosed after IUFD, and seven were diagnosed at the time of laparotomy. The two most recent cases were diagnosed with AAP preoperatively by ultrasound and MRI. Six cases were described for preoperative diagnosis. There were two cases of placenta previa, one of a bicornuate uterus, one of breech presentation, one of a combination of uterine cervical fibroids and placenta previa, and one of a combination of presentation and placental abnormality with uterine fibroids. In five cases, the placenta was removed at the time of laparotomy. Simultaneous removal of the placenta during laparotomy could not be performed because of intra-amniotic infection with a macerated fetus in an IUFD case. Among eight cases, excluding 20-week and 21-week gestation with no expectation of viable newborns, there were one male and seven female fetuses. The birth weight ranged from 1765 to 3520 g, with a median birth weight of 2241 g. Combined malformations were described in six of the seven live births. Clubfoot, torticollis, joint contracture, and bone deformity were transient because intrauterine compression quickly improved.

**Conclusion:**

In recent cases, AAP has been diagnosed by MRI and ultrasound. MRI should be performed if abdominal pregnancy is suspected. Postoperative infections may occur if the placenta is not removed at the time of delivery. We recommend placental resection with the help of an anesthesiologist, a gynecologist, a urologist, and a surgeon in the current medical environment.

## 1. Introduction

Abdominal pregnancy rarely continues to a live birth, but sometimes a live birth may occur [1–3]. Therefore, obstetricians need to have sufficient knowledge about this condition. In developed countries, women with advanced abdominal pregnancy (AAP) who have not been diagnosed preoperatively are expected to be diagnosed quickly, and the pregnant woman and the fetus will be saved. If possible, the placenta should be removed at the time of delivery of the newborn, and if difficult, it is expected to be removed in a second-look operation with minimal intraoperative bleeding. In this study, we retrospectively investigated AAP cases after 20 weeks of gestation in Japan. We investigated preoperative diagnosis of abdominal pregnancy in the second half of pregnancy before delivery and performance of placental resection at the time of delivery or at a second-look and a third-look operation.

After careful examination of the past cases, we sought to derive what is the best diagnosis and treatment choice in the current medical environment when biological tissue adhesives and electrocoagulation hemostatic devices are also available.

## 2. Methods

We searched Japana Centra Revuo Medicina [1] (Japanese PubMed) with the key word “abdominal pregnancy” from 1982 to 2021. There were 10 cases of AAP reported in the literature in which the clinical course was adequately described in the articles. We also searched articles in PubMed [4] with the key words “advanced abdominal pregnancy” and “Japan.” There was no case of AAP.

We contacted the AAP authors directly to confirm any unclear points. We reviewed complicated disease before laparotomic surgery, the presence or absence of preoperative diagnosis, the number of gestational weeks of delivery, prognosis of the newborn, the presence or absence of malformations, and the presence or absence of simultaneous excision of the placenta.

This study was approved by the Institutional Review Board and Ethics Committee of Meiwa General Hospital with application number H310115.

## 3. Results (Table 1) [5–15]

The median publication year was 1988. The maternal age ranged from 22 to 46 years, with a median age of 33 years. Eight women were primiparas, one was multipara, and one was not described. Two patients were diagnosed with abdominal pregnancy before laparotomy (cases 2 and 4), one was diagnosed after intrauterine fetal death (IUFD) (case 5), and seven were diagnosed at the time of laparotomy. Two of the patients were transferred immediately after laparotomy and abdominal wall closure at 33 weeks (case 3) and 40 weeks of gestation (case 6) to higher level medical institutions for maternal and fetal management.

In two recent cases in 2002 (cases 2 and 4), the women were diagnosed with AAP preoperatively by ultrasound and magnetic resonance imaging (MRI). In 1989, one woman was diagnosed with AAP after IUFD with the insertion of a balloon catheter in the uterine cavity under abdominal ultrasound guidance (case 5). Of the eight patients who were not diagnosed preoperatively, six were described for preoperative diagnosis. There were two cases of placenta previa (cases 8 and 10), one of a bicornuate uterus (case 1), one of breech presentation (case 6), one of a combination of uterine cervical fibroids and placenta previa (case 9), and one of a combination of presentation abnormality and placental abnormality with uterine fibroids (case 5).

The number of gestational weeks of delivery ranged from 20 to 42 weeks, with a median of 37.5 weeks. Simultaneous removal of the placenta during laparotomy was performed in five cases (cases 1, 6, 7, 8, and 10), second-look removal of the placenta was performed in four cases (case 2, 3, 4, and 5), and third-look removal of the placenta was performed in one case (case 9). There were two male and seven female fetuses within description. Two fetuses had no expectation of being viable newborns at 20 and 21 weeks of gestation (cases 7 and 9). Neonatal birth weight ranged from 1765 to 3520 g, with a median birth weight of 2241 g in the eight cases. Among these eight cases, there was one case of IUFD (case 5) and seven were live births. Simultaneous removal of the placenta during the first laparotomy was not performed in case 5 because of intra-amniotic infection with a macerated fetus. The fetus was extracted, but the placenta was not extracted due to infection. The abdominal wall was not closed, and marsupialization was performed. Second-look placental resection at 17 days postoperatively was performed combined with left salpingectomy and partial resection of the uterus.

Malformations were described in six of seven live birth cases. There were two cases of clubfoot (cases 3 and 4), two of torticollis (cases 2 and 10), one of right ankle contraction and skull deformity (case 1), and one of occipital protrusion, a small mandible, short neck and webbed neck, and mild contracture of the elbow joint and hip joint (case 8). All these conditions were transient because intrauterine compression quickly improved.

The number of medical institutions where pregnant women visited before fetal delivery was at least one or more in seven cases. One case, with 20-week tuberculous peritonitis, was not seen by a previous obstetric doctor (case 7), and two cases were not described in previous medical institutions (cases 8 and 9).

Blood transfusion was performed in four of five cases where the placenta was removed in the first surgery (cases 1, 6, 8, and 10). No blood transfusion was performed in one case at 20 weeks of gestation (case 7). Blood transfusion was also performed in a case at 21 weeks where the placenta could not be removed during the first surgery (case 9).

When placental removal was performed, including the first surgery and second-look and third-look surgeries, combined surgery was performed in six cases. The combined surgery was left salpingo-oophorectomy in three cases (cases 1, 6, and 10), left salpingectomy and partial resection of the uterus in one (case 5), supravaginal amputation of the uterus in one (case 8), and total hysterectomy in one (case 9). There were no combined resections in four cases (cases 2, 3, 4, and 7).

Common findings as shown by MRI were that the fetus, placenta, and amniotic fluid space were on the cephalic and posterior side of the normal uterus.

## 4. Discussion

Abdominal pregnancy remains a serious problem worldwide, especially in developing countries where there are limitations in diagnostic resources [4, 5, 16, 17]. Abdominal pregnancy is also difficult to detect at an early stage [6], even in countries where medical institutions are not conveniently situated, in those with large land areas, in those with poorly developed traffic transportation systems, and in those where expensive medical care is required [4, 5]. Even if AAP cases from different countries are collected, an effective management method cannot be designed. Therefore, in this study, only Japanese cases in Japan were collected (case 1–10).

Ultrasonography and MRI are required to diagnose abdominal pregnancy. Ultrasonography can only observe the fetus and surrounding organs near the probe, but MRI shows the whole trunk. Images can be reviewed later with colleagues [18]. The main implantation sites of abdominal pregnancy are the pelvic cavity near tubes and ovaries [19]. Pregnancy contents are partially buried in the pelvic cavity. Pregnancy contents are not surrounded by uterine tubes. Sliding sign is occasionally observed. Sliding sign is where the pregnancy contents are buried in the retroperitoneal tissue slide below the uterus in the pelvic cavity with pressure of a vaginal ultrasound probe. When only ultrasonography is performed, the diagnostic criteria described above are also useful in the first trimester, especially in cases where the implantation site of abdominal pregnancy is the pelvic cavity near tubes and ovaries [18].

Abdominal pregnancy is usually implanted near the ovary and fimbria of the fallopian tube. The retroperitoneum, posterior surface of the uterus, posterior lobe of the broad ligament, and adnexes are attached [19]. The organs of combined resection are usually limited to the uterus and adnexes [5, 9, 10, 12–15]. Intestinal resection and colostomy are usually not necessary. However, a surgeon or urologist should be contacted at the time of surgery.

An abdominal pregnancy may infiltrate to neighboring tissue or organs or spread far from the pelvic cavity in the early stage. In such cases, complete resection of the pregnancy contents became invasive, dangerous, and difficult. This condition resembles persistent trophoblastic disease after conservative tubal surgery, which is treated with methotrexate. However, no fetal heart-beat positive case was observed in this condition, and this finding was not observed in AAP cases [20].

Postoperative infections occur if the placenta is not removed at the time of delivery [9]. Placental resection may be attempted if the maternal anesthesia is managed properly by an anesthesiologist, and the facility is adequate for emergency blood transfusions. Biological tissue adhesives and electrocoagulation hemostatic devices are now also available. For obstetricians, it seems quite possible to remove the placenta that has been implanted in the usual Douglas fossa and adhered to the surrounding organs with the help of a gynecologist who is familiar with cervical cancer surgery, a urologist who is familiar with bladder cancer surgery, and a surgeon who is familiar with colorectal cancer surgery. It seems that it is not much difficult from the AAP placental resection to remove advanced bladder cancer, cervical cancer, and colorectal cancer.

## 5. Conclusion

In recent cases, AAP has been diagnosed by MRI and ultrasound. MRI has a wide imaging range, and it allows easy understanding of the relationship between the pregnant uterus and surrounding organs. Therefore, MRI should be performed if abdominal pregnancy is suspected.

Postoperative infections occur if the placenta is not removed at the time of delivery. We recommend placental resection with the help of an anesthesiologist, a gynecologist, a urologist, and a surgeon.

## Figures and Tables

**Table 1:** Clinical characteristics of 10 cases of abdominal pregnancy after 20 weeks of gestation reported in Japan.

Case	Authors	Age (years), gravity, parity	Weeks at diagnosis, findings	Preoperative diagnostic modality	Number of former medical institutions before birth	Preoperative diagnosis	Gestational weeks, sex, birth weight, neonatal outcome	Neonatal abnormalities	Initial operation	Second-look operation
1	Kuroda, Aragane, Miyoshi et al. [5]	34, 0G0P	37 weeks, intraoperative findings	Not diagnosed	1	Bicornuate uterus	37 weeks, female, 2504 g, alive	Contracture of the right ankle, skull deformity	Extraction of the fetus, extraction of the placenta simultaneously, left salpingo-oophorectomy, blood transfusion	No
2	Watanabe, Yoshinaga, Takami et al. [6–8]	29, 0G0P	37 weeks before the operation	MRI, US	More than 1	Abdominal pregnancy	39 weeks, female, 1978 g, alive	Torticollis	Extraction of the fetus, no blood transfusion	Second-look placental resection at 59 days postoperatively
3	Watanabe, Yoshinaga et al. [6, 7]	39, 5G1P	33 weeks, intraoperative findings, transferred soon	Not diagnosed	More than 1	Not described	33 weeks, male, 1826 g, alive	Clubfoot	Extraction of the fetus	Second-look placental resection at 84 days postoperatively
4	Watanabe et al. [6]	22, 1G0P	25 weeks, before operation	MRI, US	More than 2	Abdominal pregnancy	33 weeks, female, 1765 g, alive	Clubfoot	Extraction of the fetus	Second-look placental resection at 102 days postoperatively
5	Kajimura et al. [9]	25, 0G0P	38 weeks, after IUFD was diagnosed by US	US, insertion of a balloon catheter in the uterine cavity	more than 2	Abdominal pregnancy	38 weeks, female, 1929 g, IUFD	Grade 1 maceration (epidermis)	Extraction of the fetus, marsupialization, no blood transfusion	Second-look placental resection at 17 days postoperatively combined with left salpingectomy and partial resection of the uterus
6	Tamura et al. [10]	37, 0G0P	40 weeks, intraoperative findings, transferred soon	Not diagnosed	More than 1	Breech presentation	40 weeks, female, 3300 g, alive	None	Extraction of the fetus, extraction of the placenta simultaneously, left salpingo-oophorectomy, drainage, blood transfusion	No
7	Sekiguchi et al. [11]	36, 0G0P	20 weeks, during the operation	Not diagnosed	Tuberculous peritonitis, pregnancy not allowed, first visit at 20 weeks	Not described	20 weeks, male, 80 g, abortion due to tuberculosis	Not described	Extraction of fetus, extraction of the placenta simultaneously, no blood transfusion	No
8	Tozawa, Hamada et al. [12, 13]	34, 1G0P	38 weeks, intraoperative findings	Not diagnosed	Not described	Total placenta previa	38 weeks, female, 2850 g, alive	Occipital protrusion, small mandible, short neck, webbed neck, and mild contracture of the elbow joint and hip joint	Extraction of the fetus, extraction of the placenta simultaneously, supravginal amputation of the uterus, blood transfusion	No
9	Yonei, Tachibana et al. [14]	46, not described	21 weeks, during the operation	Not diagnosed	Not described	Uterine cervical fibroids, placenta previa	21 weeks, sex and weight not described, artificial abortion	Not described	Evacuation of pregnancy and abdominal total hysterectomy were scheduled. Extraction of the fetus, gauze tamponade in the pelvic cavity, abdominal closure, blood transfusion	Second-look gauze extraction at 3 days postoperatively and third-look placental resection at 1 year postoperatively with hysterectomy
10	Takeda et al. [15]	25, 0G0P	42 weeks, intraoperative findings	Not diagnosed	More than 1	Placenta previa	42 weeks, female, 3520 g, alive	Left torticollis	Extraction of the fetus, extraction of the placenta simultaneously, left adnectomy, blood transfusion	No

## Data Availability

The data used to support the findings of this study are available from the corresponding author upon request.
